# S-Nitrosation of Cellular Proteins by NO Donors in Rat Embryonic Fibroblast 3Y1 Cells: Factors Affecting S-Nitrosation

**DOI:** 10.1155/2011/450317

**Published:** 2011-08-17

**Authors:** Norihiro Ryuman, Nobuo Watanabe, Takao Arai

**Affiliations:** Department of Applied Biological Science, Faculty of Science and Technology, Tokyo University of Science, 2641 Yamazaki, Noda, Chiba 278-8510, Japan

## Abstract

The mechanism of protein S-nitrosation in cells is not fully understood. Using rat 3Y1 cells, we addressed this issue. Among S-nitrosothiols and NO donors tested, only S-nitrosocysteine (CysNO) induced S-nitrosation when exposed in Hanks' balanced salt solution (HBSS) and not in serum-containing general culture medium. In HBSS, NO release from CysNO was almost completely abolished by sequestering metal ions with a metal chelator without affecting cellular S-nitrosation. In contrast, L-leucine, a substrate of L-type amino acid transporters (LATs), significantly inhibited S-nitrosation. The absence of S-nitrosation with CysNO in general culture medium resulted not only from a competition with amino acids in the medium for LATs but also from transnitrosation of cysteine residues in serum albumin. Collectively, these results suggest that in simple buffered saline, CysNO-dependent S-nitrosation occurs through a cellular incorporation-dependent mechanism, but if it occurs in general culture media, it may be through an NO-dependent mechanism.

## 1. Introduction

Nitric oxide (NO) plays diverse roles in physiological processes, such as vasodilatation, host defenses against infection, and neuromodulation, some of which are mediated by the activation of the guanylate cyclase (GS)/cGMP pathway [1, 2]. Accumulating evidence suggests that NO also acts as a signaling molecule through the formation of S-nitrosothiols in proteins. S-nitros(yl)ation is a post-translational modification of proteins and low molecular weight thiols in which the nitrosonium cation attaches to the thiolate anion of a cysteine residue in proteins in a net reaction [2, 3]. Protein S-nitrosation has been shown to regulate the functions of various proteins including caspases [3, 4]. However, excessive S-nitrosation of certain proteins has been proposed as a causative event for some diseases [5–7]. More than 100 proteins have been identified to undergo S-nitrosation [3].

 Incubation of cysteine-containing proteins or low molecular weight thiols with NO donors in a simple aqueous solution under aerobic conditions yields S-nitrosothiols. The principal mechanism has been proposed to be mediated by dinitrogen trioxide (N_2_O_3_) formation as follows [8]:

(1) $$\begin{array}{cc} & \text{2NO}+{\text{O}}_{\text{2}}\to \text{2N}{\text{O}}_{2} \end{array}$$

(2) $$\begin{array}{cc} & \text{N}{\text{O}}_{\text{2}}+\text{NO}\to {\text{N}}_{2}{\text{O}}_{3} \end{array}$$

(3) $$\begin{array}{cc} & {\text{N}}_{2}{\text{O}}_{3}+\text{Protein-Cys-SH}\to \\ & \;\;\text{Protein-Cys-SNO}+{\text{N}{\text{O}}_{2}}^{-}+{\text{H}}^{+} \end{array}$$

In case of S-nitrosation of intact cells by NO, similar mechanisms are believed to occur in the hydrophobic interior of proteins or the plasma membranes [9, 10]; however, recent studies have implicated the involvement of low molecular weight amines, such as urate, as a catalyst for the reaction between N_2_O_3_ and cysteine [11, 12]. Alternatively, it is suggested that dinitrosyl iron complexes (DNIC), which are formed from NO, cytosolic chelatable iron, and thiols are responsible for S-nitrosation of cellular proteins [13]. Similarly, several mechanisms have been proposed for S-nitrosation of cells exposed to extracellular S-nitrosothiols. In particular, S-nitroso-L-cysteine (CysNO) is widely used as an “NO donor” [5, 6, 14, 15] because it spontaneously decomposes in aqueous solutions to release NO. However, CysNO has been suggested to enter the cells via L-type amino acid transporters (LATs) [16–19], thus leading to S-nitrosation of cellular proteins. In some cell types, cell surface-associated protein disulfide isomerase (PDI) has been shown to participate in the incorporation of nitroso moieties or NO from CysNO or other S-nitrosothiols into cells [20–22]. Furthermore, recent studies also proposed a novel mechanism for the transfer of nitroso moieties from S-nitrosated albumin to cells via caveolae [23]. 

In addition to the ambiguity of the mechanism, the extent of S-nitrosation that can be detected by commonly used assays, such as the biotin-switch assay or the 2,3-diaminonaphthalene (DAN) assay [24, 25], is inconsistent among various cell types and assay conditions. For example, while activation of NO synthases or exposure to exogenous NO derived from pure NO donors, such as DETA NONOate, can cause S-nitrosation of cellular proteins within 30 min in some cell types [14, 26] in other cell types these NO donors fail to induce S-nitrosation within such a short period of time [27]. Similarly, brief (<30 min) exposure to CysNO, but not GSNO, causes S-nitrosation in a wide variety of cell types [18, 28]; however, in HEK293 cells, which are very popular for molecular biology studies due to their amicability to transfection with foreign genes, GSNO can cause S-nitrosation within such short periods of time [14, 27, 29]. Moreover, in the case of spinal cord neurons, S-nitrosation is induced by GSNO and not by CysNO [27]. 

These confounding situations prompted us to investigate the factors responsible for inconsistent S-nitrosation observed in cultured cells, as well as the mechanism of S-nitrosation. We demonstrate here that among the potential nitrosating agents examined, only CysNO can induce S-nitrosation in rat embryonic fibroblast 3Y1 cells when treated in buffered saline. This mechanism is independent of the action of liberated NO or transnitrosation of cell surface proteins, but is dependent on its cellular uptake by LATs. In a cell culture medium, however, S-nitrosating activity of CysNO is suppressed not only by competition of the reaction with amino acids in the medium but also by the transnitrosation reaction with cysteine residues in serum albumin.

## 2. Materials and Methods

### 2.1. Chemicals

5,5′-Dithiobis(2-nitrobenzoic acid) (DTNB) and mercury (II) chloride (HgCl_2_) were purchased from Wako Chemicals (Osaka, Japan). L-cysteine, diethylenetriaminepentaacetic acid (DETAPAC), ethylenediaminetetraacetic acid (EDTA), Hanks' balanced salt (HBSS), L-leucine, methyl methanethiosulfonate, and N-ethylmaleimide (NEM) were purchased from Sigma-Aldrich (St. Louis, MO). Biotin-N-[6-Biotinamido)hexyl]-3′-(2′-pyridyldithio)-propionamide was purchased from Thermo Scientific (Rockford, Ill). 2,3-diaminonaphthalene (DAN), (±)-(E)-4-ethyl-2-[(E)-hydroxyimino]-5-nitro-3-hexenamide (NOR-3), and 3-morpholinosydnonimine (SIN-1) were purchased from Dojindo (Kumamoto, Japan). Horseradish peroxidase- (HRP-) conjugated antibiotin antibody was purchased from Bethyl Laboratories (Montgomery, Tex). D-cysteine was purchased from Tokyo Kasei Co. (Tokyo, Japan). All other chemicals and salts were of the highest purity available. 

CysNO and S-nitrosoglutathione (GSNO) were synthesized by mixing 0.2 M NaNO_2_ with an equal concentration of the respective thiol in HCl, followed by neutralization with NaOH [30]. The concentration of the respective nitrosothiol was determined spectrometrically using extinction coefficients *ε*_338_ = 900 M^−1^cm^−1^ and *ε*_335_ = 920 M^−1^cm^−1^ for CysNO and GSNO, respectively. Under these conditions, the yield of each nitrosothiol was more than 90%.

### 2.2. Cells and Treatment

Rat embryonic fibroblast 3Y1 cells [31] were routinely cultured in DMEM medium (Wako 041-29775) supplemented with 10% (v/v) FCS, 100 U/ml penicillin G, and 100 *μ*g/ml streptomycin under a humidified atmosphere containing 5% CO_2_ at 37°C. Experiments were performed using cells at confluence in 6-well plates or 60 mm dishes. Before exposure to the respective S-nitrosating agent, the cell culture medium was replaced with fresh 10% FCS/DMEM medium or HBSS, and the cell cultures were equilibrated in a CO_2_ incubator for 30 min. Next, the concentrated test nitrosothiols (50–250-fold final concentration) or NO donors were added to the respective culture medium, and the cells were maintained for designated time periods in a CO_2_ incubator. 

### 2.3. DAN Assay

Fluorometric detection of cellular S-nitrosothiols was performed according to the method used by Kostka and Park [25]. Briefly, after treatment, cells were washed with PBS and lysed in a lysis buffer (0.1% [w/v] SDS, 0.5% [v/v] Triton X-100, 0.5 mM EDTA, and 5 mM NEM in PBS). The lysates were divided into two parts of 200 *μ*l, and 10 *μ*l DAN in 0.8 N HCl was added to both parts. Next, to one part, 1 *μ*l of 100 mM HgCl_2_ dissolved in dimethylformamide (DMF) was added and to the other only DMF was added. After incubation at room temperature for 15 min, 10 *μ*l of 2 N NaOH was added to each part, and 200-*μ*l aliquots were transferred to the wells of a white fluorescence assay plate. Naphthotrizole fluoresecence was measured using a fluorescence plate reader (Fluoro Count, Packard) with an excitation and emission wavelength of 380 nm and 460 nm, respectively. Mercury-dependent fluorescence was converted to S-nitrosothiol concentrations by a standard curve made of serially diluted NaNO_2_ in the same lysis buffer without HgCl_2_ treatment. 

### 2.4. Biotin-Switch Assay

Protein S-nitrosation was detected by the biotin-switch assay as previously described [24]. Briefly, after treatment, cells were lysed in a lysis buffer composed of HNE buffer (250 mM HEPES [pH 7.8], 1 mM EDTA, and 0.1 mM neocuproine), 10% (w/v) SDS, and 10% (v/v) MMTS in DMF at a ratio of 9 :1 : 0.2, respectively. Samples were then incubated at 50°C with occasional vortexing for blocking any free thiols. Proteins were precipitated with 70% acetone at −20°C, and precipitates were collected by centrifugation at 8000 ×g for 5 min. The pellets were washed in a similar manner three times with 70% acetone. The pellets were dissolved in HEN buffer containing 0.25 mg/ml biotin-HPDP and 1% SDS, briefly sonicated, and then mixed with sodium ascorbate to obtain a final concentration of 1 mM. Following 1-h incubation at room temperature, proteins sedimented were washed by the acetone precipitation method as described above. The pellets were dissolved in 0.1% SDS in PBS. Equal amounts of the biotinylated proteins were resolved by SDS-PAGE using 10% or 6%–16.5% gradient acrylamide gel, and then transferred to a polyvinylidene difluoride membrane (Immobirone, Millipore). After blocking the membrane with 1% (w/v) skim milk in PBS containing 0.05% (v/v) Tween 20, the membrane was probed with HRP-conjugated antibiotin antibody. The biotinylated proteins were visualized using an ECL reagent (GE Healthcare), and the signal was recorded using LAS3000 image analyzer (Fuji Film Co., Japan). 

### 2.5. Other Assays

NO released from nitrosothiol degradation in the media was aerobically measured using an NO electrode (Apollo 1000, World Precision Instrument) at 37°C. The device was calibrated according to the manufacturer's instructions.

S-alkylation of the cysteine residues in BSA was performed using NEM. Defatted BSA in PBS (30 mg/ml) was incubated with NEM (15 mM) at room temperature for 1 h, and excess NEM was thoroughly removed by dialysis against PBS. The thiol content of BSA before and after NEM treatment was evaluated by the DTNB method using GSH as a standard. After NEM treatment, the number of free thiols decreased from 0.37 to less than 0.02 SH per BSA molecule.

 Protein concentration was determined by the BCA method (Pierce Biotechnology) with BSA as standard. 

### 2.6. Statistics

Data are expressed as mean ± SEM. The Student's *t*-test or one-way analysis of variance followed by the Tukey's test was used for appropriate statistical analyses.

## 3. Results and Discussion

### 3.1. All Potential S-Nitrosating Agents Caused S-Nitrosation in 3Y1 Cell Lysates

**Figure 1:**
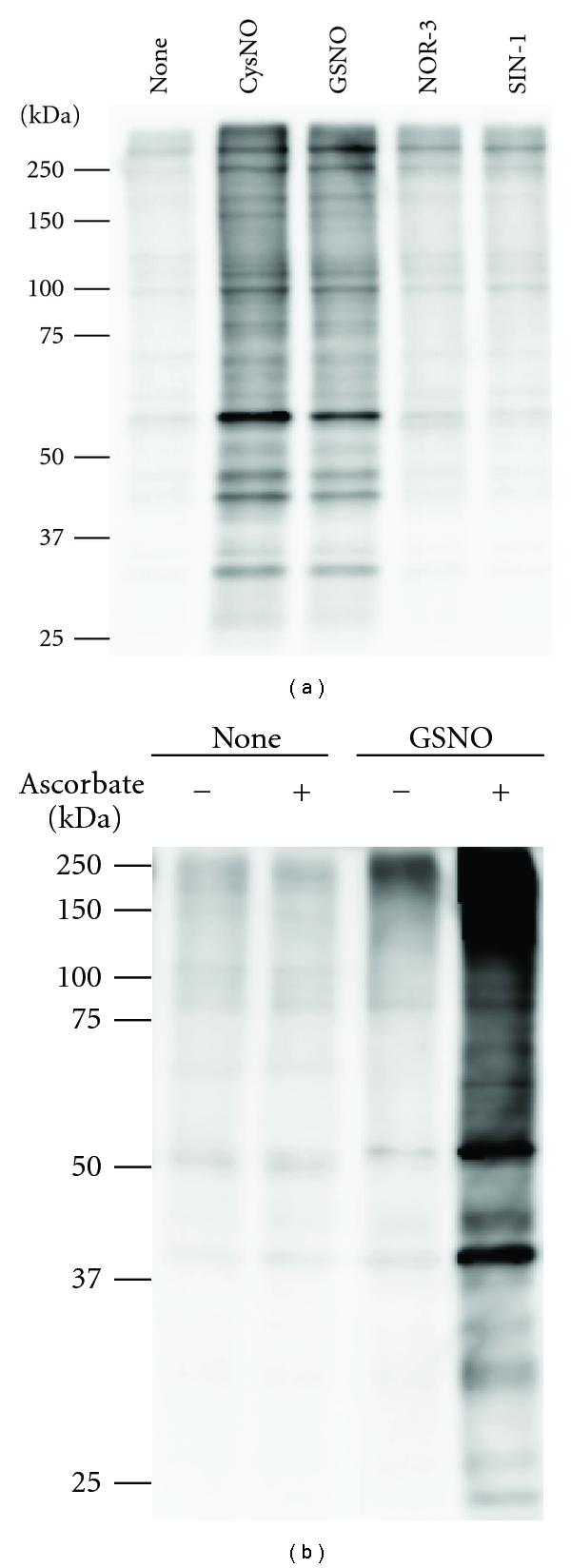
Protein S-nitrosation induced by potential S-nitrosating agents in 3Y1 cell lysates. (a) Detection of S-nitrosated proteins in cell lysate treated with potential S-nitrosating agents. 3Y1 cell lysates prepared in PBS containing 0.5 mM EDTA and 0.5% Triton X-100 were incubated with CysNO (200 *μ*M), GSNO (200 *μ*M), NOR-3 (200 *μ*M), or SIN-1 (1 mM) at 37°C for 30 min. S-nitrosated proteins were detected by the biotin-switch assay. (b) Specificity of the biotin switch assay. 3Y1 cells lysates were incubated with or without GSNO (500 *μ*M), and the resulting S-nitrosated proteins were assessed by biotin-switch assay as above, but with or without ascorbate in the biotinylation step. A 10% gel was used for this confirmation experiment. Therefore, the resolution of S-nitrosated protein bands was different from that in all other experiments in which 6.0%–16.5% gradient gels were used.

In an aqueous solution, S-nitrosation of thiols can be induced by various NO donors and S-nitrosothiols, as well as conditions where NO and O_2_^−^ are generated together [32]. The ability of these potential S-nitrosating agents/conditions to S-nitorosate proteins in 3Y1 cell lysates was compared. CysNO and GSNO were used as the test S-nitrosothiols, NOR-3 as the NO donor, and SIN-1 as the NO/O_2_^−^ cogenerator. At a neutral pH and 37°C, NOR-3 and SIN-1 both have a half-life of approximately 30 min [33, 34]. The cell lysates were incubated with CysNO (200 *μ*M), GSNO (200 *μ*M), NOR-3 (200 *μ*M), or SIN-1 (1 mM) at 37°C for 30 min, and S-nitrosated proteins were detected by the biotin-switch assay. As shown in Figure 1(a), GSNO and CysNO potently S-nitrosated almost the same array of proteins to a similar extent. NOR-3 and SIN-1 (when tested at a higher concentration of 1 mM) could also cause S-nitrosation of many proteins. However, the extent of S-nitrosation caused by either compound was considerably less than that induced by either GSNO or CysNO. The specificity of S-nitrosated proteins detected by the biotin-switch assay was confirmed. Without ascorbate in the biotinylation step, the number of S-nitrosated protein bands decreased to almost untreated control level, indicating that our biotin-switch assay detected S-nitrosation (Figure 1(b)).

### 3.2. Only CysNO Can S-Nitrosate Live 3Y1 Cells in HBSS but Not in Culture Medium

**Figure 2:**
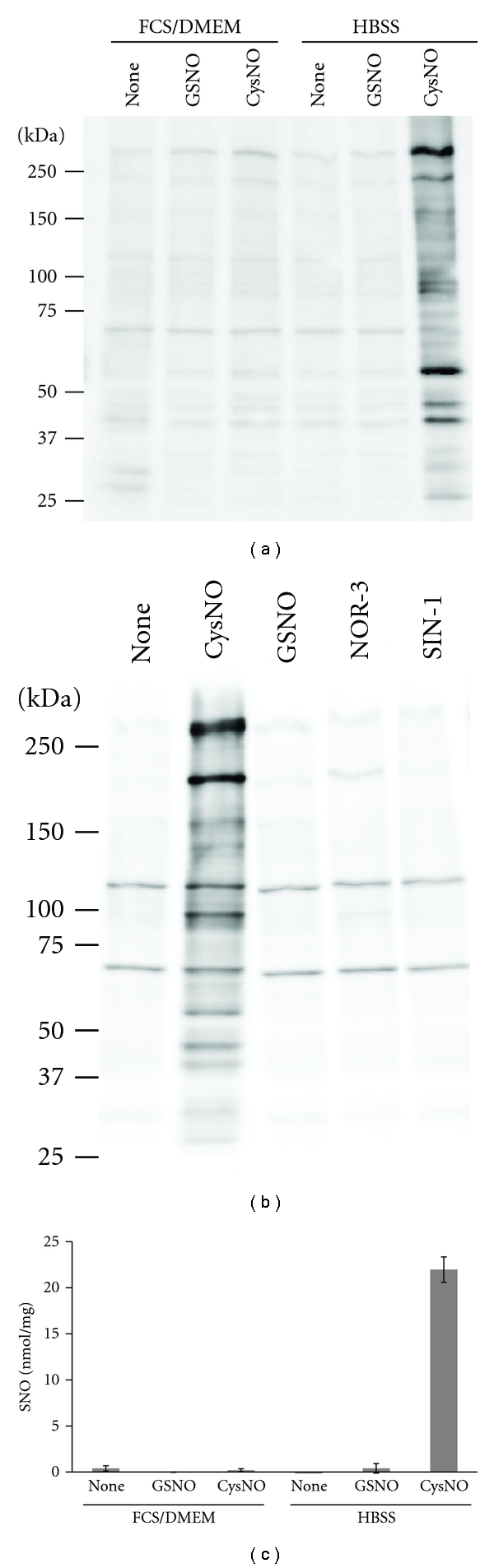
Protein S-nitrosation induced by exposure to potential S-nitrosating agents in intact 3Y1 cells. (a) Comparison of the efficiencies of potential S-nitrosating agents to S-nitrosate 3Y1 cells. Cells maintained in HBSS were treated with CysNO (200 *μ*M), GSNO (200 *μ*M), NOR-3 (200 *μ*M), or SIN-1 (1 mM) for 30 min. S-nitrosated proteins were detected by the biotin-switch assay. (b and c) Effects of media on S-nitrosation efficiency of S-nitrosothiols. Cells cultured in 10% FCS/DMEM and HBSS were treated with GSNO (1 mM) or CysNO (1 mM) for 30 min, and S-nitrosated proteins were detected by (b) the biotin-switch assay and (c) the DAN assay. The blots shown in (a) and (b) are representative results from several experiments with similar results. Data in (c) are shown as mean ± SEM of three to four independent assays.

Next, the extent of protein S-nitorosation occurring in intact cells exposed to each S-nitrosating agent was examined. To minimize reactions and/or interactions (i.e., transnitrosation) of the nitrosating agents with media components and to evaluate the primary effects of the test agents, exposure time was set to 30 min. When cells maintained in HBSS were incubated with CysNO (200 *μ*M), GSNO (200 *μ*M), NOR-3 (200 *μ*M), or SIN-1 (1 mM), only CysNO resulted in S-nitrosation of various proteins in these cells (Figure 2(a)). Under these treatment conditions, 1 mM CysNO resulted in maximum S-nitrosation while 200 *μ*M CysNO resulted in approximately 50% of the maximum level (data not shown). However, when the same treatment was performed in a general culture medium (10% FCS/DMEM), none of these agents caused S-nitrosation in these cells (data not shown). In 10% FCS/DMEM, even 1 mM CysNO was unable to induce protein S-nitrosation (Figure 2(b)). Furthermore, 1 mM GSNO failed to induce S-nitrosation regardless of the media (Figure 2(b)). The DAN assay can detect not only S-nitrosated proteins but also cytosolic S-nitrosated low molecular weight thiols such as GSNO. When examined using the DAN assay, the prominent S-nitrosating ability of CysNO over GSNO was observed in HBSS (each at 1 mM), but not in 10% FCS/DMEM (Figure 2(c)). These results suggest that S-nitrosation efficiency in intact 3Y1 cells in culture depends not only on the nitrosating agent but also on the incubation medium. 

### 3.3. Cellular Uptake of CysNO, rather than NO Liberation, Is Responsible for S-Nitrosation in Intact 3Y1 Cells

**Figure 3:**
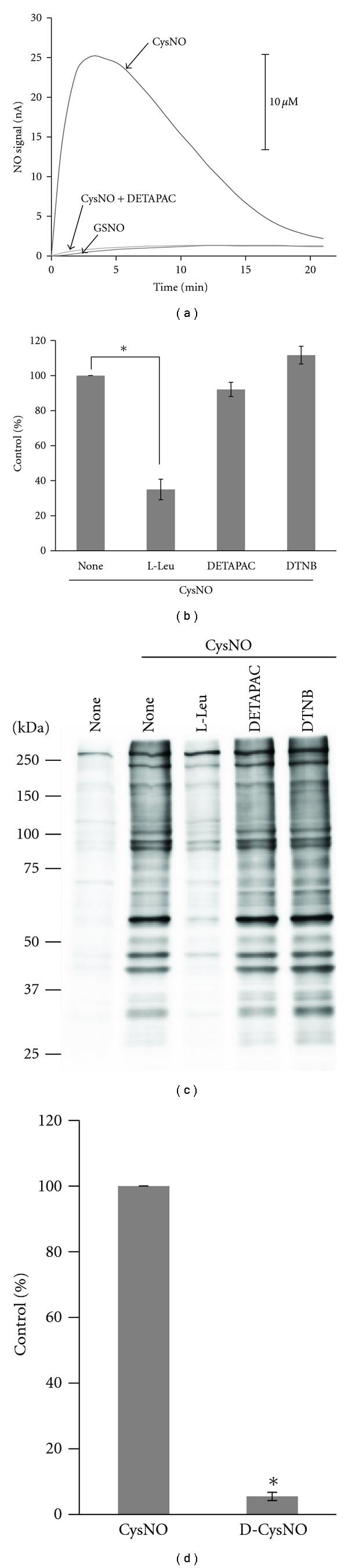
Effects of DETAPAC, DTNB, and L-leucine on CysNO-induced S-nitrosation in 3Y1 cells. (a) NO release from S-nitrosothiols in HBSS. CysNO or GSNO was diluted in HBSS to a final concentration of 200 *μ*M without or with DETAPAC (0.5 mM). NO concentration in the medium was aerobically monitored at 37°C using an NO electrode. The trace shown is a representative result. (b and c) Effects of DETAPAC, DTNB, and L-leucine on CysNO-induced S-nitrosation in 3Y1 cells. Cells in HBSS were treated with CysNO (200 *μ*M) for 30 min in the absence or presence of DETAPAC (0.5 mM), L-leucine (1 mM), and DTNB (100 *μ*M), which were added 20 min before the addition of CysNO. Protein S-nitrosation was detected using (b) the DAN assay and (c) the biotin-switch assay. The blot shown is a representative result. (d) The efficiency of CysNO- and D-CysNO-induced S-nitrosation, each at 200 *μ*M, was measured by the DAN assay as in (b). S-nitrosothiol levels in cells treated with CysNO alone (labeled “none” in (b)) were taken as 100% (12 ± 2 nmol SNO/mg protein), and the values are expressed as the mean ± SEM of six to eight independent experiments in (b) and from three independent experiments in (d). *P < 0.001.

 Next, the S-nitrosating ability of CysNO in 3Y1 cells was investigated to determine if S-nitrosation was caused by NO released by CysNO or some other mechanism. Thus, we measured the concentration of NO that was derived from the spontaneous degradation of CysNO (200 *μ*M) in the media using an NO electrode. When diluted with HBSS at 37°C, CysNO immediately liberated NO that reached a peak 3 min after dilution and declined gradually thereafter (Figure 3(a)). In contrast, only marginal amount of NO was released from GSNO under identical conditions. Similar marked difference was observed in the stability of CysNO and GSNO in HBSS when Hg^2+^-cleavable SNO moieties were measured by Saville-Griess assay [35]; CysNO decayed with a half-life of 10 min, whereas essentially no decay was observed for GSNO after 2 h (data not shown). The stability of CysNO in a solution depends on the amount of ionic transition metal impurities, such as iron [36]. To examine the involvement of any transition metal ions, the effect of the metal chelator DETAPAC on NO release was measured. DETAPAC presence drastically inhibited NO release from CysNO, indicating that a metal-catalyzed breakdown reaction is responsible for NO liberation from CysNO in HBSS under the assay conditions used. 

To clarify whether NO derived from CysNO degradation in the medium was responsible for S-nitrosation of cellular proteins, we examined the effect of DETAPAC on S-nitrosation by CysNO in cells maintained in HBSS. DETAPAC presence did not affect the extent of S-nitrosation as evaluated by the DAN assay (Figure 3(b)). Evaluation of individual proteins by the biotin-switch assay also demonstrated that DETAPAC failed to inhibit protein S-nitrosation (Figure 3(c)). Since the peak of NO levels derived from CysNO was approximately 3 min, some proteins might transiently undergo S-nitrosation [37] by CysNO through an NO-dependent mechanism. However, because the S-nitrosating ability of NO (NOR-3) or NO/O_2_^∙−^ (SIN-1) was very poor (Figures 1(a) and 2(b)), the formation of S-nitrosated proteins by NO derived from CysNO could be small in number and amount, if any. Meanwhile, for a majority of S-nitrosated proteins that are stably present after 30 min, the results thus far clearly rule out the involvement of NO as an intermediate in cellular S-nitrosation by CysNO. 

LAT [18, 28, 38] and thiol-containing plasma membrane proteins, including cell surface-associated PDI [20–22], have been suggested to be involved in S-nitrosation. The addition of L-leucine (1 mM), a competitive substrate of LAT [39], inhibited the extent of CysNO-induced S-nitrosation by more than 60%, as evaluated by the DAN assay (Figure 3(b)). The biotin-switch assay also demonstrated a similar inhibitory effect of leucine on S-nitrosation of individual proteins (Figure 3(c)). DTNB, due to its hydrophilic nature, can mask only cell-surface cysteine sulfhydryl groups through the formation of S–S bonds with thionitrobenzoic acids. Pretreatment with DTNB for 30 min masked 12 nmol cysteine/mg of proteins (data not shown). However, pretreatment with DTNB had no effect on the extent or profile of any S-nitrosated proteins (Figures 3(b) and 3(c)). These results suggest that CysNO-induced S-nitrosation of cellular proteins is largely mediated by CysNO uptake through LAT, rather than interactions with or transnitrosation of cell surface thiol-containing proteins. 

To further ascertain LAT involvement in S-nitrosation of these cells by CysNO, S-nitrosating activity of CysNO stereoisomer, S-nitroso-D-cysteine (D-CysNO), was evaluated. Previous studies have demonstrated that D-CysNO cannot be incorporated through LAT [39]. The kinetics of NO release from D-CysNO in HBSS, as measured using a NO electrode, was essentially superimposable on that of L-CysNO (data not shown). The extent of S-nitrosation induced by D-CysNO was less than 10% of that induced by the L-isomer (Figure 3(d)), thus confirming the predominant role of LAT in CysNO-dependent S-nitrosation in this cell line.

### 3.4. Transnitrosation of BSA and Competition with Amino Acids for LAT Are Responsible for the Suppression of CysNO-Mediated S-Nitrosation in the Cell Culture Medium

**Figure 4:**
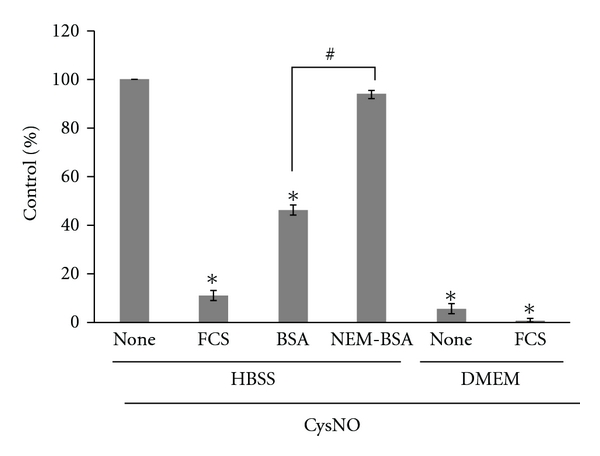
Effects of FCS, BSA, and NEM-BSA on CysNO-mediated S-nitrosation in 3Y1 cells. Cells maintained in HBSS or DMEM in the absence or presence of FCS (10%), BSA (3 mg/ml), or NEM-treated BSA (3 mg/ml) were treated with CysNO (200 *μ*M) for 30 min. The levels of S-nitrosated proteins were measured by the DAN assay, and the value for cells treated with CysNO in HBSS alone (none) was taken as 100% (12 ± 2 nmol/mg protein). Values are mean ± SEM of three to four independent assays. *P < 0.001 versus none; ^#^ *P* < 0.001 versus BSA.

CysNO failed to S-nitrosate cells in the cell culture medium (10% FCS/DMEM; Figure 2). Therefore, we next investigated the mechanism responsible for absence of CysNO-induced S-nitrosation in the cell culture medium (Figure 4). CysNO- (200 *μ*M)-induced S-nitrosation in cells maintained in HBSS was significantly suppressed by the addition of FCS (10%), suggesting that the serum contains factors that inhibit S-nitrosation. Since the principal proteinaceous component of serum is BSA, whose concentration is approximately 3 mg/ml in 10% serum, its effect was measured. BSA supplementation (3 mg/ml) in HBSS inhibited CysNO-induced S-nitrosation, although not as significantly as FCS at this concentration. BSA has 35 cysteine residues of which 34 are involved in intramolecular disulfide bridges and half of the one remaining is blocked by a free cysteine via a disulfide bond [40]. To evaluate the role of the free sulfhydryl group of BSA in its suppressive action on S-nitrosation, the effect of NEM-treated BSA was measured. NEM treatment decreased the number of free sulfhydryl groups in BSA from 0.37 to less than 0.02 (see Section 2). Interestingly, when the same concentration of NEM-treated BSA was supplemented in HBSS, no inhibitory effects were observed, suggesting that free sulfhydryl groups in BSA play a role in the inhibition of CysNO-dependent S-nitrosation, possibly by receiving the nitroso group in a transnitrosation reaction. 

In the absence of FCS, CysNO can weakly S-nitrosate cells in DMEM, although the extent of this was less than 10% of that observed in HBSS (Figure 4), suggesting that the DMEM basal medium also contains inhibitory factors. However, when cells were treated with a high CysNO concentration (1 mM), the suppressive effect of DMEM decreased by 50% (data not shown), indicating the competitive nature of inhibition. These results suggest that the suppression of S-nitrosation in 10% FCS/DMEM was due to the presence of neutral amino acids in the basal medium that act as competitive substrates for LAT, in addition to the interaction of CysNO with free cysteine residues in BSA.

### 3.5. Implication for the Mechanism of S-Nitrosation by CysNO

CysNO is widely used as an “NO donor” to S-nitrosate cells [5, 6, 14, 15], although S-nitrosation was not associated with the action of NO under our assay conditions. Among various the mechanisms for S-nitrosation by extracellular S-nitrosothiols [17, 18, 20–23, 28, 38, 41], Hogg et al. [17, 18, 41] and Whorton et al. [28, 38] have shown that in several cell types, including erythrocytes, endothelial cells, smooth muscle cells, and epithelial cells, S-nitrosation of cellular proteins involves LAT-mediated CysNO uptake. Through this study, we add embryonic fibroblast 3Y1 cells to this growing cell inventory. With regard to this, absence of S-nitrosation by GSNO is consistent with the role of LAT as the primary route for S-nitrosothiol uptake in this cell line. The present results, along with previous results reported by the above-mentioned authors [18, 28, 38, 41], warn that cautious interpretation is necessary for results that indicate cellular S-nitrosation by CysNO. Specifically, if CysNO can cause S-nitrosation in some cell types in a general cell culture medium [5, 6, 14, 15], some parts of the mechanism may be NO-dependent, such as the formation of N_2_O_3_ and DNIC as intermediates, because LAT-mediated uptake could be suppressed under these conditions. In fact, the amount of NO released from CysNO in 10% FCS/DMEM was attenuated, but it persisted possibly due to transnitrosation of Cys residue in serum albumin (data not shown). However, when cellular S-nitrosation is induced in a simple buffered saline solution, the primary mechanism could largely depend on LAT-mediated cellular uptake. Therefore, the use of D-CysNO is probably preferable when CysNO is employed as the NO donor to nitrosate cells. 

### 3.6. Conclusion

The present study demonstrates that in buffered saline only CysNO can cause significant levels of protein S-nitrosation in rat 3Y1 cells when exposed for a short period of time. The mechanism is not driven by the actions of NO derived from CysNO degradation in the medium, but by the incorporation of CysNO through LAT. However, in the cell culture medium, LAT-mediated CysNO uptake is almost completely prevented by the presence of competitive amino acids and transnitrosation of serum albumin. Care should be taken when interpreting the biological effects of CysNO in cells because some of its effects may be due to NO donation and others by protein S-nitrosation and that the overall effect seen will depend to some extent on the particular cell type studied and the incubation conditions.
